# Clinical study of vacuum-assisted excision of deep breast nodules via the retromammary space

**DOI:** 10.3389/fsurg.2025.1697308

**Published:** 2025-11-06

**Authors:** Yuan-Yu Ma, Hai-Na Xin, Bin Ren

**Affiliations:** 1 School of Clinical Medicine, Shandong Second Medical University, Weifang, Shandong, China; 2 General Surgery and Breast Surgery Department, Weifang Maternal and Child Health Hospital, Weifang, Shandong, China; 3 Department of Gastrointestinal and Anorectal Surgery I, Affiliated Hospital of Shandong Second Medical University, Weifang, Shandong, China

**Keywords:** minimally invasive rotary excision, retromammary approach, vacuum-assisted excision, deep breast nodules, postoperative complications

## Abstract

**Objective:**

To compare the therapeutic efficacy of retromammary-space vacuum-assisted excision (VAE) and non-retromammary VAE for deep breast nodules ≤3 cm in maximum diameter, providing a clinical basis for selecting the optimal surgical approach.

**Methods:**

A retrospective analysis was conducted on the clinical data of 162 patients who underwent minimally invasive surgery for deep breast nodules at Weifang Maternal and Child Health Hospital between May 2022 and November 2024. Among them, 81 patients received retromammary-space VAE, while another 81 underwent non-retromammary VAE. Based on nodule size, the retromammary-space VAE group was further divided into two subgroups: the 0–2 cm group (*n* = 41, 0 < nodule ≤ 2 cm) and the 2–3 cm group (*n* = 40, 2 cm < nodule ≤ 3 cm). The two groups were compared in terms of operative time, intraoperative blood loss, number of punctures, and incidence of postoperative complications. Subgroup analysis within the retromammary-space VAE group further compared these parameters between the two size-based subgroups.

**Results:**

Compared with non-retromammary VAE, retromammary-space VAE demonstrated statistically significant advantages (*P* < 0.05) in key parameters, including shorter operative time, less intraoperative blood loss, fewer punctures, and a lower incidence of postoperative complications. Subgroup analysis revealed that the 0–2 cm group had shorter operative time and less blood loss than the 2–3 cm group (*P* < 0.05), while the number of punctures did not differ significantly between groups (*P* > 0.05).

**Conclusion:**

Retromammary-space VAE for deep benign breast nodules can effectively reduce intraoperative bleeding, shorten operative time, and lower the incidence of complications such as pectoralis muscle injury and hematoma. This technique allows precise puncture positioning beneath the nodule, minimizes the number of punctures, and maintains this advantage regardless of nodule size, demonstrating promising clinical applicability and potential for wider adoption.

## 1 Introduction

Benign breast nodules are among the most common benign breast diseases (BBD) (1), and their incidence has shown a steadily increasing trend in recent years (2). According to the 2024 Global Cancer Statistics, the detection rate of breast nodules in adult women has reached as high as 75% (3), and this figure continues to rise. Due to their specific anatomical location, deep-seated breast nodules have long been a clinical focus in terms of diagnosis and treatment. Such nodules may not only affect the normal physiological function of the breast but also carry a potential risk of malignant transformation (4). One study reported that patients with a history of BBD have an increased risk of breast cancer regardless of whether atypical hyperplasia is present (5), indicating that a history of BBD is an independent risk factor for breast cancer. Furthermore, multiple studies have demonstrated that a family history of benign breast lesions in first-degree relatives, genetic mutations (BRCA1, BRCA2), and abnormal estrogen levels are also high-risk factors for breast cancer (6). Therefore, early and accurate qualitative diagnosis is of great significance for selecting appropriate surgical strategies and improving patient prognosis.

At present, the treatment of deep breast nodules mainly includes traditional open surgery and minimally invasive procedures (7). For deep nodules (≤3 cm) requiring surgical intervention, traditional approaches often result in greater trauma, slower recovery, and altered chest wall contour, thereby affecting quality of life. Minimally invasive vacuum-assisted excision (VAE), with its advantages of precise ultrasound-guided localization, minimal trauma, and superior cosmetic outcomes, has gained increasing favor (8). Theoretically, retromammary-space VAE can reduce glandular injury and postoperative complications compared with non-retromammary VAE; however, comparative data on key indicators such as operative time and complication rates for deep nodules of different sizes remain limited. This study analyzes the clinical data of retromammary-space VAE and non-retromammary VAE to evaluate their efficacy and safety in treating deep breast nodules of varying sizes.

## 2 Materials and methods

### 2.1 General information

This study included 162 patients who underwent surgery for deep breast nodules at Weifang Maternal and Child Health Hospital between May 2022 and November 2024. According to the minimally invasive surgical approach, patients were divided into the retromammary-space VAE group (*n* = 81) and the non-retromammary VAE group (*n* = 81). The retromammary-space VAE group was further subdivided based on nodule size into the 0–2 cm group (*n* = 41) and the 2–3 cm group (*n* = 40). Preoperative evaluation of nodules was performed according to the 5th edition of the BI-RADS classification.

Inclusion criteria: (1) breast nodules classified as BI-RADS 3–4a with a maximum diameter ≤3 cm; (2) patients with multiple unilateral breast nodules who underwent excision of only one nodule; (3) color Doppler ultrasound indicating that the nodule was located in the deep glandular region of the breast, with a distance of <0.5 cm from the pectoralis major muscle; (4) postoperative pathology confirming benign lesions; (5) patients who provided informed consent and signed the consent form.

Exclusion criteria:

(1) Pathological diagnosis of malignancy; (2) severe coagulation dysfunction; (3) multiple nodule excisions performed in one breast; (4) inability to avoid major blood vessels or main lactiferous ducts under ultrasound guidance; (5) long-term use of contraceptives or hormone drugs, or current pregnancy, lactation, or preconception period; (6) presence of breast implants, allergy to local anesthetics, or concomitant systemic diseases.

This study was approved by the Ethics Committee of Weifang Maternal and Child Health Hospital (Approval No. KY-2025-06).

### 2.2 Methods

#### 2.2.1 Surgical equipment

A color Doppler ultrasound system (M8 Super; Mindray Medical, Shenzhen, China) and a rotary breast lesion vacuum-assisted biopsy system (DK-B-MS; Xishan Technology, Chongqing, China) were used in this study.

#### 2.2.2 Pre-treatment preparations

Prior to surgery, all patients underwent comprehensive preoperative evaluation to exclude surgical contraindications, and written informed consent was obtained. Ultrasound was used preoperatively to locate the puncture site and mark the surface projection of the nodule, including its position and size, with a straight line drawn along the planned trajectory. For the retromammary-space VAE group, the puncture site was preferably selected at the outer margin of the breast or approximately 5 cm away from the nodule if necessary, while for the non-retromammary VAE group, the puncture site was chosen directly above or near the nodule according to the shortest and most accessible route.

#### 2.2.3 Surgical methods

##### 2.2.3.1 Retromammary-space VAE group

A tumescent local anesthetic solution was prepared by mixing 10 mL of 2% lidocaine hydrochloride, 50 mL of normal saline, and 0.1 mL of 1:1,000 epinephrine. Patients were positioned appropriately, and the skin was disinfected with povidone-iodine. Local infiltration anesthesia was first administered at the puncture site using a syringe, followed by subcutaneous superficial anesthesia. Under ultrasound guidance, additional anesthetic was injected incrementally while advancing the needle until the tip reached the retromammary space beneath the nodule. A sudden reduction in injection resistance indicated correct placement. A larger volume of tumescent anesthetic was then infused to expand the retromammary space, preparing the site for excision.

**Figure 1:**
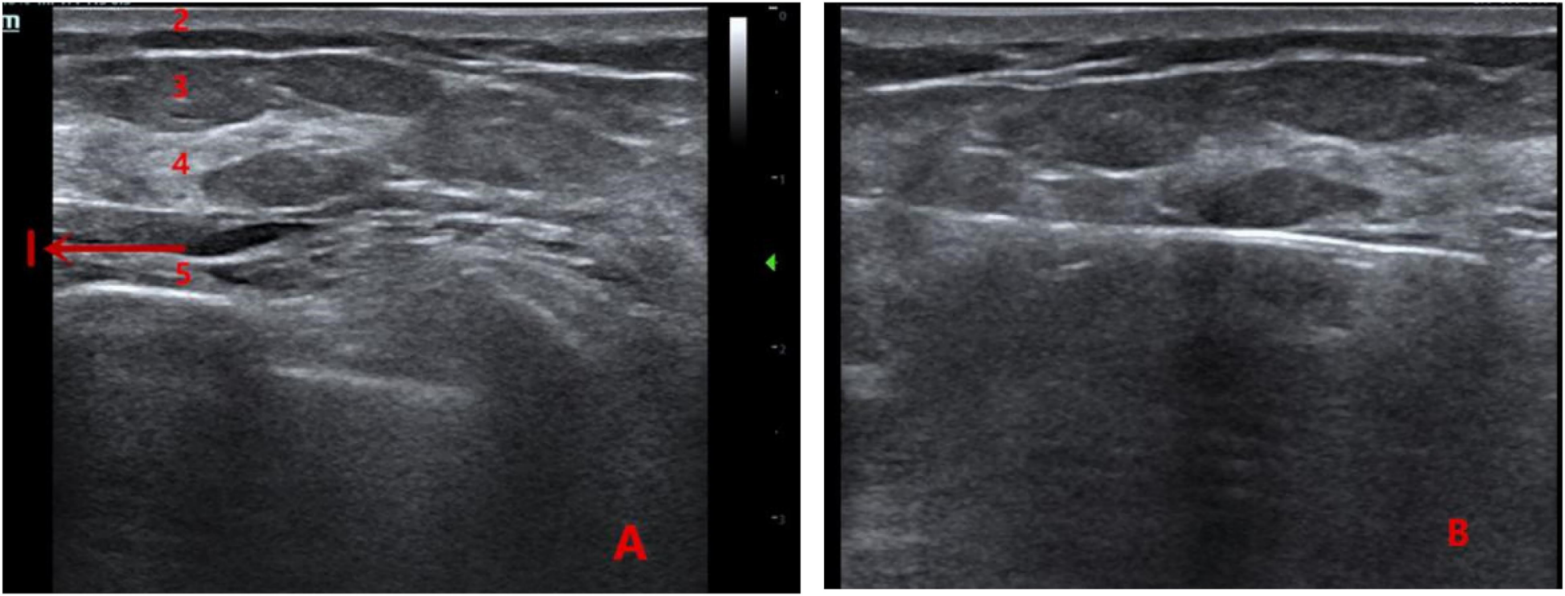
**(A)** Ultrasound image showing the anatomical layers of the breast (1: anechoic area formed by local anesthetic swelling in the retromammary space; 2: skin layer; 3: fat layer; 4: glandular layer; 5: pectoralis muscle layer). **(B)** Intraoperative view of the retromammary-space VAE procedure.

A 4–5 mm incision was made at the puncture site. Under ultrasound guidance, the rotary excision needle was inserted through the shortest distance of glandular tissue into the expanded retromammary space beneath the nodule. The needle was adjusted so that the cutting groove was positioned beneath the nodule. The lesion was excised using a fan-shaped rotational movement, with continuous adjustment of the needle under real-time ultrasound guidance. Vacuum aspiration was used to remove residual blood. After confirming the absence of residual tissue using a crosswise inspection technique, the needle was withdrawn, residual blood was expressed from the cavity, and the incision was covered with a sterile dressing. Firm compression was applied over the incision and cavity for 5 min, and an elastic bandage was applied for at least 48 h. The rotary excision needle was flushed with 250 mL of normal saline, and the specimen was collected (Figure 1).

##### 2.2.3.2 Non-retromammary VAE group

Patients were positioned and disinfected in the same manner. Local infiltration anesthesia was applied at the puncture site, followed by infiltration along the subcutaneous layer, needle tract, and around the nodule. A 4–5 mm incision was made at the puncture site. Under real-time ultrasound guidance, the rotary excision needle was advanced through the breast tissue to the base of the nodule, and excision was performed in the same manner as in the retromammary-space VAE group.

#### 2.2.4 Definition of complications

1. Pectoralis muscle injury: Presence of muscle tissue identified in the excised specimen postoperatively.
2. Nodule residue: Solid echogenic area detected by ultrasound at the original nodule site 3 months after surgery, showing positional and morphological correlation with the preoperative nodule.
3. Hematoma: Ultrasound performed on postoperative day 1 revealing a low- to anechoic area ≥2 cm in maximum diameter at the original nodule site, with a palpable firm area and marked tenderness.

#### 2.2.5 Follow-up observation

##### 2.2.5.1 Intraoperative indicators

Intraoperative blood loss (mL) was calculated as the volume of fluid collected in the postoperative drainage container (mL) minus 250 mL. Operative time, intraoperative blood loss, and number of punctures (defined as the number of attempts required to accurately position the rotary excision needle behind the nodule before excision) were compared between groups.

##### 2.2.5.2 Postoperative complication indicators

The incidence of postoperative complications, including residual cavity hematoma and pectoralis muscle injury, was compared between groups. Ultrasound was performed at 3 months postoperatively to evaluate for residual tissue.

### 2.3 Statistical analysis

Data were analyzed using SPSS version 27.0. Measurement data conforming to a normal distribution were expressed as mean ± standard deviation (*x¯* ± *s*), and categorical data were expressed as composition ratios or percentages (%). Comparisons of measurement data between groups were performed using the independent-samples t-test, while comparisons of categorical data were conducted using the chi-square (*χ*^2^) test or Fisher's exact test. Multivariate analysis was performed using logistic regression. A *P* value < 0.05 was considered statistically significant.

## 3 Results

### 3.1 Comparison of general data

The comparison of general clinical characteristics (age, tumor length, lesion location, and lesion size) between the non-retromammary VAE group and the retromammary-space VAE group is shown in Table 1. No statistically significant differences were observed between the two groups (*n* refers to the number of patient cases).

**Table 1:** Comparison of baseline characteristics between patients in the two surgical approach groups.

Variable	Non-retromammary VAE group (*n* = 81)	Retromammary-space VAE group (*n* = 81)	*t*/*x*^2^	*p*
Age (years)	37.91 ± 10.89	38.20 ± 9.84	−0.174	0.862
Tumor length (cm)	1.68 ± 0.65	1.67 ± 0.64	0.158	0.875
Location of the lump				
Left breast (*n*, *n*%)	36 (44.44%)	48 (59.26%)	3.56	0.059
Right breast (*n*, *n*%)	45 (55.56%)	33 (40.74%)		
Size of the lump				
<2 cm (*n*, *n*%)	41 (50.62%)	41 (50.62%)	0	1
>2 cm (*n*, *n*%)	40 (49.38%)	40 (49.38%)		

### 3.2 Comparison of intraoperative indicators

Compared with the control group, the observation group had significantly lower intraoperative blood loss, fewer punctures, and shorter operative time, with statistically significant differences between the groups (*P* < 0.05; Table 2).

**Table 2:** Comparison of surgical outcomes between the two approach groups $\left(\bar{x}\pm s\right)$.

Index	Non-retromammary VAE group (*n* = 81)	Retromammary-space VAE group (*n* = 81)	*t*	*p*
Intraoperative blood loss (mL)	11.77 ± 2.47	9.40 ± 2.33	6.284	<0.001
Operative time (min)	20.57 ± 4.27	17.63 ± 3.38	4.856	<0.001
Number of punctures	2.53 ± 0.91	1.31 ± 0.46	10.77	<0.001

In the subgroup analysis of the retromammary-space VAE group, the 0–2 cm subgroup showed significantly less intraoperative blood loss and shorter operative time compared with the 2–3 cm subgroup (*P* < 0.05), while the difference in the number of punctures was not statistically significant (*P* > 0.05), as shown in Table 3.

**Table 3:** Comparison of surgical outcomes in the retromammary-space VAE group $\left(\bar{x}\pm s\right)$.

Index	0–2 cm (*n* = 41)	2–3 cm (*n* = 40)	*t*	*p*
Intraoperative blood loss (mL)	8.07 ± 1.79	10.75 ± 2.02	−6.305	<0.001
Operative time (min)	15.24 ± 2.12	20.08 ± 2.59	−9.208	<0.001
Number of punctures	1.34 ± 0.48	1.27 ± 0.45	0.641	0.523

### 3.3 Comparison of postoperative complications

The incidence of postoperative complications in the retromammary-space VAE group was 4.93%, including 1 case of pectoralis muscle injury, 1 case of residual nodule at 3-month follow-up, and 2 cases of postoperative hematoma. In the non-retromammary VAE group, the incidence was 14.81%, including 4 cases of pectoralis muscle injury, 3 cases of residual nodule at 3-month follow-up, and 5 cases of postoperative hematoma. The difference between the two groups was statistically significant (Table 4).

**Table 4:** Comparison of complications between the two surgical approach groups (*n*).

Complication items	Non-retromammary VAE group (*n* = 81)	Retromammary-space VAE group (*n* = 81)	*x* ^2^	*p*
Pectoralis muscle injury	4 (4.94%)	1 (1.23%)	0.825^a^	0.364
Residual nodule	3 (3.70%)	1 (1.23%)	0.256^a^	0.613
Hematoma	5 (6.17%)	2 (2.47%)	0.597^a^	0.440
Incidence of complications	12 (14.81%)	4 (4.93%)	4.438	0.035

a Adjusted chi-square test was used.

In the retromammary-space VAE group, the incidence of postoperative complications was 2.4% in the 0–2 cm subgroup, including 1 case of hematoma, and 7.5% in the 2–3 cm subgroup, including 1 case of pectoralis muscle injury, 1 case of residual nodule at 3-month follow-up, and 1 case of hematoma. Although the 0–2 cm subgroup had a lower incidence of complications than the 2–3 cm subgroup, the difference was not statistically significant (*P* > 0.05; Table 5).

**Table 5:** Comparison of complications in the retromammary-space VAE group (*n*).

Complication items	0–2 cm (*n* = 41)	2–3 cm (*n* = 40)	*x* ^2^	*p*
Pectoralis muscle injury	0	1 (2.5%)	—	0.494^a^
Residual nodule	0	1 (2.5%)	—	0.494^a^
Hematoma	1 (2.4%)	1 (2.5%)	—	1.000^a^
Incidence of complications	1 (2.4%)	3 (7.5%)	0.290	0.590^b^

a Fisher's exact test was used.

b Adjusted chi-square test was used.

### 3.4 Logistic multivariate analysis of the retromammary-space VAE group and the non-retromammary VAE group

The results of the multivariate analysis showed that, after adjusting for confounding factors such as age, lesion location (left/right), lesion size, and ultrasound classification, the retromammary-space VAE group demonstrated advantages over the non–retromammary VAE group in terms of intraoperative blood loss (OR = 0.44), operative time (OR = 0.55), and number of punctures (OR = 0.02), indicating that the retromammary-space VAE procedure offers superior safety and efficiency (*P* < 0.05), as shown in Table 6.

**Table 6:** Logistic multivariate analysis.

Index	*B*	SE	Wals	OR (95%CI)	*P*	OR (95%CI)
Intraoperative blood loss	−0.465	0.133	12.238	0.63 (0.48, 0.82)	<0.001	0.44 (0.29, 0.66)
Operative time	−0.063	0.072	0.783	0.94 (0.82, 1.08)	<0.001	0.55 (0.41, 0.75)
Number of punctures	−2.618	0.451	33.734	0.07 (0.30, 0.18)	<0.001	0.02 (0.01, 0.12)

### 3.5 Logistic multivariate analysis between the 0–2 cm and 2–3 cm subgroups in the retromammary-space VAE group

The results of the multivariate analysis showed that, after adjusting for confounding factors such as age, lesion location (left/right), and ultrasound classification, the 0–2 cm subgroup demonstrated advantages over the 2–3 cm subgroup in terms of intraoperative blood loss (OR = 2.39) and operative time (OR = 2.54), indicating that smaller nodules are associated with higher safety and efficiency (*P* < 0.05), as shown in Table 7.

**Table 7:** Logistic multivariate analysis.

Index	*B*	SE	Wals	OR (95%CI)	*P*	OR (95%CI)
Intraoperative blood loss	0.679	0.218	9.697	1.97 (1.29, 3.02)	<0.001	2.39 (1.40, 4.07)
Operative time	0.879	0.221	15.772	2.41 (1.56, 3.71)	<0.001	2.54 (1.55, 4.14)
Number of punctures	0.779	1.029	0.574	0.45 (2.18, 16.38)	0.54	2.00 (0.22, 18.27)

### 3.6 Comparison of postoperative pathology

Specimens from both surgical approach groups were sent for pathological examination, and the pathological findings are presented in Table 8.

**Table 8:** Pathological findings of specimens from the two surgical approach groups.

Postoperative pathology	Retromammary-space VAE group	Non-retromammary VAE group	Total
Fibroadenoma	56	53	67.28%
Breast cyst	10	10	12.35%
Mammary adenosis	11	12	14.20%
Benign phyllodes tumor	4	4	4.94%
Intraductal papilloma	0	2	1.23%

## 4 Discussion

The etiology of breast nodules is mainly related to environmental factors, changes in lifestyle, and endocrine hormone disorders (9). Open surgery is associated with significant trauma and bleeding in patients (10), and improper surgical procedures may lead to poor postoperative recovery and decreased quality of life (11). In current clinical practice, ultrasound-guided minimally invasive rotary excision is a commonly used treatment for breast nodules (12). Under ultrasound guidance, the rotary needle is placed beneath the nodule so that the probe, nodule, and needle groove are in the same plane, perpendicular to the chest wall, and the nodule is excised from bottom to top and from the center toward both sides (13). Multiple studies have demonstrated that ultrasound-guided minimally invasive rotary excision achieves favorable outcomes in the removal of deep breast nodules. Studies have shown that patients with breast nodules located at a depth ≥10 mm from the skin who underwent ultrasound-guided minimally invasive VAE were classified as the deep-excision group. The results demonstrated that the deep-excision group had smaller maximum nodule diameters, shorter operative times, shorter incision lengths, and faster postoperative wound healing compared with the traditional open surgery group (14). Another study divided patients undergoing ultrasound-guided Mammotome VAE into superficial and deep minimally invasive excision groups based on the depth of the nodule from the skin surface. It was found that intraoperative blood loss, operative time, and hospital stay did not differ significantly between the two minimally invasive groups, and both were markedly better than the traditional surgery group, further confirming that this technique achieves favorable surgical outcomes in the removal of deep breast nodules (15). In addition to these findings, this study selected retromammary-space VAE rather than traditional open segmental excision for deep breast nodules measuring 2–3 cm in maximum diameter because of the minimally invasive nature and superior cosmetic outcomes of VAE. In this study, nodules of this size were completely excised using the VAE technique, with a low incidence of postoperative complications. The retromammary space has also been widely utilized in other breast procedures. For example, in endoscope-assisted breast cancer surgery via an axillary approach, a concealed incision along the axillary fold takes advantage of the anatomical features of the retromammary space, providing a broader operative field and facilitating breast-conserving surgery or subcutaneous gland resection with implant-based reconstruction, thereby avoiding anterior chest wall scarring (16–18).

In this study, retromammary-space VAE was applied for the excision of deep breast nodules of varying sizes. The results showed that this technique demonstrated advantages over non–retromammary VAE across several key indicators. Specifically, operative time was shorter, which not only reduced the potential risks associated with prolonged anesthesia but also improved operating room efficiency. Moreover, the reduction in intraoperative blood loss was beneficial for postoperative recovery (19), facilitating faster physical rehabilitation. The number of punctures, an important indicator of surgical precision (20), was lower in the retromammary-space VAE group, and this advantage was not affected by nodule size. Accurate puncture and localization beneath the nodule minimized unnecessary blind punctures and reduced tissue trauma to the breast, thereby lowering the risk of postoperative complications. These findings highlight the technical superiority of retromammary-space VAE, as its precise localization technique effectively enhances surgical accuracy and safety. A lower incidence of postoperative complications was also a notable advantage of this approach. Complications such as pectoralis muscle injury and hematoma can cause additional pain, prolong recovery, and potentially affect the cosmetic appearance and function of the breast. By optimizing the surgical path and operating technique, retromammary-space VAE effectively reduced the occurrence of such complications. Further subgroup analysis revealed that nodules measuring 0–2 cm were associated with less intraoperative blood loss and shorter operative time compared with those measuring 2–3 cm, suggesting that nodule size influences these two indicators. Smaller nodules may be easier to manipulate and less disruptive to surrounding tissues, resulting in reduced bleeding and shorter procedures. However, no significant difference was observed in the number of punctures between the two subgroups, indicating that the precise localization capability of retromammary-space VAE remains consistent regardless of nodule size, maintaining stable puncture accuracy in the treatment of nodules of different dimensions.

When performing non-retromammary VAE for the excision of deep benign breast nodules, the puncture needle must pass through the glandular layer to reach the posterior aspect of the lesion. In young patients with dense and firm breast tissue, this procedure is technically challenging. If the breast is not well stabilized, the puncture needle tip may cause the gland to shift during insertion, leading to the disappearance of the lesion from the ultrasound image and necessitating multiple puncture attempts for accurate localization, thereby increasing tissue trauma (21, 22). In contrast, the retromammary-space VAE utilizes tumescent anesthesia applied to the subcutaneous and retromammary spaces without injecting the solution into the glandular tissue. This approach avoids the formation of “pseudo-lesion” artifacts, facilitates precise identification and excision of the nodule, and shortens the operation time. By accessing the retromammary space created by the tumescent anesthesia, the puncture needle enters a wider, less resistant area that is not impeded by the firm glandular tissue. This allows smoother rotary excision with reduced damage to breast lobules and enables accurate positioning beneath the lesion, thereby achieving better *en bloc* removal from the posterior aspect. Consequently, the overall excision rate is relatively higher with this approach (23).

However, postoperative complications in the retromammary-space VAE group included one case of pectoralis major muscle injury, one case of residual lesion, and two cases of hematoma. Anatomically (24), the retromammary space lies between the deep layer of the superficial fascia and the pectoralis major fascia. Although this area contains relatively few blood vessels and nerves and its loose connective tissue facilitates surgical manipulation, its close proximity to the pectoralis major muscle increases the risk of muscle injury. The single case of pectoralis major injury observed in the 2–3 cm subgroup may be related to the narrowing or collapse of the retromammary space during negative-pressure cutting and suction when removing a larger lesion. In addition, irregularly shaped and deeply located nodules pose challenges for ultrasound visualization and require a high level of surgical skill. Finally, since hemostasis after minimally invasive excision relies primarily on compression, poor patient compliance—such as excessive or premature movement of the ipsilateral upper limb—can predispose to postoperative hematoma formation. At present, strategies for preventing various intraoperative and postoperative complications of vacuum-assisted excision, including pneumothorax, postoperative bleeding, hematoma, and residual lesions (25), continue to provide valuable guidance for clinical practice and for refining this technique.

In summary, retromammary-space VAE for the treatment of deep benign breast nodules can effectively reduce intraoperative blood loss and operative time, as well as lower the incidence of complications such as pectoralis muscle injury and hematoma, with more pronounced benefits in the treatment of smaller nodules. In addition, this technique reduces the number of punctures required, enables precise localization beneath the nodule, and maintains this advantage regardless of nodule size, indicating its potential clinical value. However, this study has certain limitations. It was a retrospective, single-center study with a relatively small sample size and non-randomized grouping, which may introduce selection bias. The postoperative follow-up period was only 3 months, so the long-term outcomes, such as nodule recurrence, remain unclear. Moreover, the local anesthetic solution used for tumescent anesthesia contained epinephrine, which may have potential hemostatic effects, possibly confounding the observed reduction in blood loss in the retromammary-space VAE group. Further studies are needed to verify these findings.

## Data Availability

The original contributions presented in the study are included in the article/Supplementary Material, further inquiries can be directed to the corresponding authors.
